# Anemia of chronic disease: Evaluating iron markers and bone marrow findings

**DOI:** 10.6026/973206300223576

**Published:** 2026-06-30

**Authors:** Preeti Nigotia, Madhur Gupta, Abhishek Yadav, Lakshya Pruthi

**Affiliations:** 1 Department of General Medicine, SRVS Medical College Shivpuri, Madhya Pradesh, India; 2 Department of Biochemistry, N.K.P. Salve Institute of Medical Sciences & Research Centre and Lata Mangeshkar Hospital, Nagpur, Maharashtra, India; 3 Department of Community Medicine, Atal Bihari Vajpayee Medical College, Vidisha, Madhya Pradesh, India; 4 Department of Internal Medicine, David Tvildiani Medical University, Georgia

**Keywords:** Anemia of chronic disease (ACD), iron metabolism, ferritin, soluble transferrin receptor (sTfR), bone marrow iron, hepcidin

## Abstract

Anemia of chronic disease (ACD) is a common condition associated with chronic inflammation, characterized by disordered iron metabolism and impaired erythropoiesis. This study evaluated iron metabolism markers and correlated them with bone marrow iron stores in 100 patients with anemia in chronic disease settings. Parameters including serum iron, ferritin, total iron-binding capacity (TIBC), transferrin saturation (TSAT) and soluble transferrin receptor (sTfR) were analyzed alongside bone marrow iron grading using Perls' Prussian blue staining. Most patients demonstrated low serum iron and TSAT with normal to elevated ferritin levels, while increased sTfR in some cases suggested coexisting iron deficiency. Data shows that integrating serum ferritin and sTfR with bone marrow iron grading provides a more reliable framework for distinguishing anemia of chronic disease from coexisting iron deficiency, supporting a dual biochemical-morphological approach over single-marker evaluation in routine clinical practice.

## Background:

Anemia of chronic disease (ACD), also known as anemia of inflammation, is frequently encountered in routine clinical practice, particularly in patients suffering from chronic infections, autoimmune disorders, malignancies and other long-standing illnesses. It is characterized by impaired iron utilization and reduced erythropoiesis, largely driven by inflammatory mechanisms [1, 2]. A key factor involved in this process is hepcidin, a regulatory hormone that plays an important role in maintaining iron balance. Increased hepcidin levels during inflammation inhibit ferroportin activity, thereby reducing intestinal iron absorption and trapping iron within macrophages [1, 3]. As a result, even when total body iron stores are adequate, its availability for erythropoiesis becomes limited. This explains why ACD differs from iron deficiency anemia (IDA), where there is an actual depletion of iron stores [4]. In addition to altered iron metabolism, inflammatory cytokines also contribute to decreased red cell production and reduced erythrocyte lifespan, further aggravating anemia [2, 5]. Therefore, ACD represents a combination of impaired iron handling and suppressed bone marrow response rather than a simple deficiency state. From a diagnostic perspective, laboratory parameters play an essential role. Serum ferritin is often normal or elevated in ACD due to its nature as an acute-phase reactant, while it is typically low in IDA [4]. However, interpretation of ferritin alone may be misleading in inflammatory conditions. Hence, additional markers such as transferrin saturation (TSAT), total iron-binding capacity (TIBC) and soluble transferrin receptor (sTfR) are commonly used to better assess iron status [6]. Bone marrow iron staining with Prussian blue continues to be considered the gold standard for evaluating iron stores, especially in cases where biochemical findings do not provide a clear diagnosis [6]. Therefore, it is of interest to identify iron metabolism markers and correlate them with bone marrow findings in patients with anemia of chronic disease in a tertiary care setting.

## Materials and Methods:

This prospective observational study was conducted in the Department of Pathology of a tertiary care center over a period of 12 months. A total of 100 adult patients clinically suspected to have anemia associated with chronic diseases were included in the study.

## Inclusion criteria:

[1] Patients aged ≥18 years

[2] Hemoglobin <13 g/dL in males and <12 g/dL in females

[3] Patients with clinically diagnosed chronic inflammatory conditions (infection, autoimmune disease, malignancy, or chronic systemic illness)

[4] Patients with normocytic or mildly microcytic anemia

## Exclusion criteria:

[1] History of acute blood loss

[2] Blood transfusion within the last 3 months

[3] Patients receiving iron supplementation

[4] Known cases of hemolytic anemia

[5] Pregnant women

## Laboratory investigations:

[1] Complete blood count (CBC) using an automated hematology analyzer

[2] Serum iron estimation

[3] Serum ferritin measurement

[4] Total iron-binding capacity (TIBC)

[5] Transferrin saturation (TSAT) calculation

[6] Soluble transferrin receptor (sTfR) levels (where indicated)

## Bone marrow examination:

Bone marrow aspiration was performed in selected cases under aseptic precautions. Iron stores were assessed using Perls' Prussian blue staining and graded accordingly.

## Statistical analysis:

The collected data were analyzed using SPSS version 25. Continuous variables were expressed as mean ± standard deviation and categorical variables as percentages. Pearson correlation test was applied to assess the relationship between serum ferritin and bone marrow iron grading. A p-value <0.05 was considered statistically significant.

## Results:

The study enrolled a total of 100 patients diagnosed with anemia associated with chronic diseases, providing a sufficiently representative sample for meaningful clinicopathological analysis. As depicted in Table 1, the study population had a mean age of 48.6 ± 15.2 years, reflecting a predominantly middle-aged cohort, with a slight male preponderance in the sex distribution (56 males versus 44 females). The most common underlying etiology contributing to anemia of chronic disease (ACD) in this cohort was chronic infection, accounting for 35% of cases (Table 1), which is consistent with the well-established pathophysiological role of persistent inflammatory states in suppressing erythropoiesis and dysregulating iron homeostasis. This demographic profile underscores the heterogeneous nature of ACD and its predilection for individuals burdened by long-standing systemic illnesses. The hematological findings summarized in Table 2 revealed a mean hemoglobin level of 9.2 ± 1.3 g/dL across the study population, indicative of mild-to-moderate anemia consistent with the expected severity range in ACD. The mean corpuscular volume (MCV) of 82.5 ± 6.1 fL and mean corpuscular hemoglobin (MCH) of 27.1 ± 3.2 pg both fall within or marginally below the lower limits of normal, collectively pointing toward a normocytic or borderline microcytic, normochromic anemia pattern. This morphological pattern is characteristic of ACD, wherein iron is sequestered within reticuloendothelial stores rather than being unavailable in absolute terms, thereby limiting its incorporation into developing erythroid precursors without producing the frank microcytosis typically observed in true iron deficiency anemia. The relatively preserved red cell indices further support the diagnosis of functional iron deficiency superimposed on a chronic inflammatory background. The iron profile of the study population, presented in Table 3, revealed a mean serum iron level of 38.4 ± 10.2 μg/dL, which is notably reduced relative to normal reference ranges, reflecting the characteristic hypoferremia of ACD driven by hepcidin-mediated sequestration of iron within macrophages and hepatocytes. In contrast, the mean serum ferritin level was markedly elevated at 320 ± 150 ng/mL (Table 3), a finding that distinguishes ACD from true iron deficiency anemia, wherein ferritin levels are typically low. The elevated ferritin in this context reflects both increased iron storage and its role as an acute phase reactant upregulated during chronic inflammatory states. The total iron inding capacity (TIBC) was reduced at 210 ± 40 μg/dL (Table 3), further corroborating the ACD pattern, as TIBC typically decreases in inflammatory conditions due to reduced transferrin synthesis, in contrast to the elevated TIBC seen in iron deficiency anemia. Transferrin saturation (TSAT) was correspondingly low at 15 ± 5% (Table 3), confirming restricted iron availability for erythropoiesis despite adequate or excess total body iron stores. The soluble transferrin receptor (sTfR) level of 2.8 ± 0.9 mg/L was mildly elevated, reflecting increased erythroid demand for iron at the cellular level and providing a useful marker for distinguishing functional from absolute iron deficiency in this population. Bone marrow iron grading, considered the gold standard for assessing total body iron stores, revealed a predominantly iron-replete pattern in this cohort, as shown in Table 4. The majority of patient's demonstrated moderate (2+) iron stores, accounting for 45% of cases, while marked (3+) iron stores were observed in 40% of patients (Table 4). Mild (1+) iron stores were present in 10% of cases, and complete absence of stainable iron was noted in only 5% of the study population (Table 4). This distribution confirms that the overwhelming majority of ACD patients in this study harbored adequate to excess bone marrow iron, reinforcing the concept that the anemia in these patients is driven not by absolute iron depletion but by pathological iron sequestration and impaired iron utilization secondary to chronic inflammatory signaling. The small proportion of patients with absent or mild iron stores may represent cases with a coexisting component of true iron deficiency, highlighting the diagnostic complexity encountered in clinical practice. Figure 1 (see PDF) illustrates the correlation between serum ferritin levels and bone marrow iron grading across the study population. The positive correlation depicted in Figure 1 (see PDF) confirms that rising serum ferritin values correspond with progressively higher grades of bone marrow iron stores, validating the utility of serum ferritin as a surrogate marker for tissue iron stores in the context of ACD. However, given ferritin's dual role as both a storage protein and an acute phase reactant, this correlation must be interpreted cautiously in patients with active inflammatory or infectious conditions, where ferritin may be disproportionately elevated relative to actual iron stores. Nevertheless, Figure 1 (see PDF) supports the use of serum ferritin as a clinically practical and reasonably reliable indicator of bone marrow iron status in this patient population, particularly when interpreted alongside other iron metabolism parameters. Figure 2 (see PDF) provides a schematic representation of the characteristic iron metabolism pattern observed in anemia of chronic disease, integrating the laboratory findings described above into a coherent pathophysiological framework. The pattern illustrated in Figure 2 (see PDF) comprising low serum iron, low TIBC, low transferrin saturation, elevated ferritin, and preserved or increased bone marrow iron stores collectively defines the hallmark biochemical signature of ACD, distinguishing it from iron deficiency anemia on one end of the spectrum and iron overload disorders on the other. This characteristic pattern, as depicted in Figure 2 (see PDF), arises primarily from hepcidin-driven internalization and retention of ferroportin on macrophages and enterocytes, effectively trapping iron within storage compartments and rendering it unavailable for erythropoiesis despite its physical abundance. The integration of all iron metabolism markers as visualized in Figure 2 (see PDF) serves as a practical diagnostic template for clinicians evaluating patients with anemia in the setting of chronic systemic disease.

## Discussion:

The findings of the present study demonstrate a clear alteration in iron metabolism among patients with anemia of chronic disease, which can largely be attributed to the effects of inflammation on iron homeostasis. Similar mechanisms have been described in previous studies, where inflammatory cytokines lead to iron sequestration and reduced availability for erythropoiesis [7]. In our study, serum ferritin levels were found to be elevated in most patients. This finding is expected, as ferritin behaves as an acute- phase reactant and increases during inflammatory states [8]. However, this also highlights a diagnostic limitation, as elevated ferritin levels may mask underlying iron deficiency when interpreted in isolation. A reduction in serum iron levels and transferrin saturation was also observed, indicating decreased circulating iron despite adequate stores. This finding is consistent with earlier reports suggesting that iron becomes trapped within the reticuloendothelial system during inflammation [9]. In addition, total iron-binding capacity (TIBC) was reduced, which can be explained by decreased transferrin production in inflammatory conditions [8, 9]. The role of soluble transferrin receptor (sTfR) was particularly useful in the present study. Since it is less affected by inflammation, it provides a more reliable indicator of cellular iron demand and helps differentiate ACD from iron deficiency anemia [6, 9]. Bone marrow examination showed increased iron stores in the majority of cases and a good correlation was observed with biochemical parameters. Although invasive, bone marrow iron assessment still remains a dependable method, especially in situations where laboratory findings are inconclusive [7]. Current clinical recommendations also emphasize the use of multiple parameters rather than relying on a single marker for diagnosis. A combined approach improves accuracy and helps guide appropriate management strategies [10]. Overall, the findings of this study suggest that no single parameter is sufficient for diagnosing anemia of chronic disease. Instead, a combination of iron markers along with bone marrow evaluation, when required, provides a more reliable and clinically useful assessment.

## Conclusion:

Data shows that anemia of chronic disease is associated with characteristic alterations in iron metabolism, including reduced serum iron, decreased transferrin saturation and normal to elevated ferritin levels, along with adequate or increased bone marrow iron stores. Although serum ferritin correlates with marrow iron, its interpretation requires caution in inflammatory conditions. Soluble transferrin receptor serves as a useful adjunct marker in identifying coexisting iron deficiency. Thus, a combined evaluation of biochemical parameters and bone marrow findings provides a more accurate and reliable approach for the diagnosis and differentiation of anemia of chronic disease, thereby aiding in appropriate clinical management.

## Figures and Tables

**Table 1:** Demographic profile

**Parameter**	**Value**
Total patients	100
Mean age	48.6 ± 15.2 years
Male : Female	56:44:00
Most common cause	Chronic infection (35%)

**Table 2:** Hematological parameters

**Parameter**	**Mean ± SD**
Hemoglobin	9.2 ± 1.3 g/dL
MCV	82.5 ± 6.1 fL
MCH	27.1 ± 3.2 pg

**Table 3:** Iron profile

**Parameter**	**Mean ± SD**
Serum Iron	38.4 ± 10.2 μg/dL
Ferritin	320 ± 150 ng/mL
TIBC	210 ± 40 μg/dL
TSAT	15 ± 5 %
sTfR	2.8 ± 0.9 mg/L

**Table 4:** Bone marrow iron stores

**Grade**	**Percentage**
Absent (0)	5%
Mild (1+)	10%
Moderate (2+)	45%
Marked (3+)	40%
